# What is mood? A computational perspective

**DOI:** 10.1017/S0033291718000430

**Published:** 2018-02-26

**Authors:** James E Clark, Stuart Watson, Karl J Friston

**Affiliations:** 1 Newcastle University, Newcastle Upon Tyne, UK; 2 Northumberland Tyne and Wear NHS Foundation Trust, Newcastle Upon Tyne, UK; 3 University College London, London, UK

## Abstract

The neurobiological understanding of mood, and by extension mood disorders, remains elusive despite decades of research implicating several neuromodulator systems. This review considers a new approach based on existing theories of functional brain organisation. The free energy principle (a.k.a. active inference), and its instantiation in the Bayesian brain, offers a complete and simple formulation of mood. It has been proposed that emotions reflect the precision of – or certainty about – the predicted sensorimotor/interoceptive consequences of action. By extending this reasoning, in a hierarchical setting, we suggest mood states act as (hyper) priors over uncertainty (i.e. emotions). Here, we consider the same computational pathology in the proprioceptive and interoceptive (behavioural and autonomic) domain in order to furnish an explanation for mood disorders. This formulation reconciles several strands of research at multiple levels of enquiry.

## The current predicament

Mood disorders are heterogeneous and complex and depend upon the interplay of several neuromodulator systems and genetic and epigenetic factors (Hirschfeld, 2000; Holsboer, 2000; Nestler & Carlezon, 2006; Nutt *et al.* 2007; Dowlati *et al.* 2010; Möhler, 2012). As a result, current approaches to diagnosis and classification of mood disorders suffer several shortcomings (Nesse & Stein, 2012), and advances in understanding the underlying neurobiology have been slow. We argue that progress may be facilitated by an appreciation of the dynamic and self-organising nature of neurobiological systems (Seth, 2013; Fotopoulou, 2015; Clark *et al.* 2016).

## The brain is a generative organ

Traditional hypotheses propose that the neurobiological underpinnings of a variety of disorders arise from structural or functional abnormalities in the brain consequent on a combination of environmental stress and genetic vulnerabilities (Videbech & Ravnkilde, 2004; Ota & Duman, 2013; Duman, 2014). Similarly, it has been argued that pharmacotherapy may work via its effects on neurogenesis and synaptic plasticity (Santarelli *et al.* 2003; Anacker *et al.* 2011; Castrén & Hen, 2013) and that stressors may leave an indelible scar on key neurobiological systems that disrupts their structure and/or function. In this sense, the traditional hypothesis is that brain abnormalities develop throughout the lifespan and result in the onset and maintenance of psychopathology.

However, modern accounts of brain function reject the classical notion of the brain as a passive organ in favour of theories of embodiment and self-organisation (Friston, 2010; Seth, 2013; Corlett & Fletcher, 2014). Specifically, recent theoretical accounts propose that the brain works towards an equilibrium in which its environment is rendered predictable; i.e. surprise is minimised and uncertainty resolved (Friston, 2009). Essentially, the brain embodies a generative model that encodes prior beliefs about sensory input and their causes. This model generates predictions which are tested against actual input to produce prediction errors (surprise). These prediction errors are then used by the brain to revise its model of the world. In so doing, it updates its predictions and minimises prediction error (Friston, 2010).

On this view, prior beliefs about the world, as discussed above, are represented in terms of probability distributions. The sufficient statistics for these distributions may be labelled as ‘expectation’ and ‘precision’ and, if the brain embodies such priors, it follows that they must be represented in its physical activity and anatomy. It is thought that expectations (and subsequent predictions) are encoded by synaptic activity, while precision, or uncertainty, is encoded by the extent to which this activity is attenuated or amplified (Friston & Kiebel, 2009), e.g. through careful synaptic efficacy or gain control. In current predictive coding formulations of the Bayesian brain, expectation and prediction error units are thought to occupy deep and superficial pyramidal layers of cortex, respectively (Friston & Kiebel, 2009; Bastos *et al.* 2012; Shipp *et al.* 2013); such that predictions generated, from expectations, at one level of the cortical hierarchy descend to form prediction errors in superficial layers of the level below. These prediction error units then send ascending signals to update expectations. Crucially, the precision or confidence placed in prediction errors is then associated with the synaptic gain or efficacy of superficial pyramidal cells – that itself depends upon interactions with inhibitory interneurons and modulatory neurotransmission. This is a brief description of hierarchical predictive coding. In what follows, we look more closely at the back story to predictive coding; namely, free energy minimisation and allostasis.

## The free energy principle

The brain, like other biological systems, seeks to maintain its physiological (and psychological) state in the face of a constantly changing internal and external environment and must therefore minimise entropy over external states (where entropy is a mathematical measure of uncertainty or expected surprise). Directly computing surprise is intractable, but by appealing to variational principles, we can calculate an upper boundary on surprise, namely free energy, which systems will (or will appear to) minimise (Friston *et al.* 2006). Given that surprise is the inverse of model evidence, if the brain is minimising a free energy bound on surprise, it is necessarily trying to maximise the sensory evidence for its model of the world and is inherently self-evidencing (Hohwy, 2016). Under some simplifying assumptions, one can equate surprise with (precision weighted) prediction error. In brief, the brain can minimise (precision weighted) prediction error in three ways. First, expectations can be updated (by changing neuronal activity) so that predictions provide a better explanation for sensory inputs (Friston *et al.* 2006). Alternatively, the brain can change the world or the way it is sampled (by engaging motor and autonomic reflexes) so that sensations fall into line with predictions. This provides a simple explanation for behaviour, which becomes the fulfilment of predicted (proprioceptive and somatosensory) motor sensations. Finally, both of these processes (perception and action) can be nuanced by optimising the precision of prediction errors. In cognitive neuroscience, this optimisation has been framed in terms of attention and attenuation. In other words, attending to a sensory stream corresponds to increasing its sensory precision through appropriate synaptic gain control. Conversely, sensory attenuation corresponds to the reduction of precision by attending away from or ignoring the consequences of one's own action.

The active minimisation of free energy is known as *active inference*, which means the brain can selectively sample from data that concur with its current expectations (Friston *et al.* 2011; Pezzulo, 2012). In the interoceptive domain, the resolution of prediction errors through autonomic reflexes provides a simple account of homoeostasis. This formulation can be extended by appealing to hierarchical generative models such that predictions at higher levels pre-empt the need for homoeostasis [e.g. in a hypoglycaemic state, by attenuating the precision of prediction errors reporting hypoglycaemia, we can suspend the reflex mobilisation of glucose and act on the world (by eating) to fulfil and maintain higher level predictions] (Pezzulo *et al.* 2015). This hierarchical minimisation of prediction errors allows allostatic control over homoeostatic reflexes (Sterling & Eyer, 1988; McEwen, 1998; Ramsay & Woods, 2014). Note that the balance between *allostasis* and *homoeostasis* depends on attenuating interoceptive prediction errors, which will be an important theme in what follows. In this way, a self-regulating embodied loop of circular causality is constructed in which the brain constructs the external environment (and internal milieu) it expects to encounter, which in turn reinforces its predictions (Seth, 2014; Barrett & Simmons, 2015).

## Free energy and emotion

Recent theoretical arguments have converged on the idea that emotional states reflect changes in the uncertainty about the somatic consequences of action (Joffily & Coricelli, 2013; Wager *et al.* 2015; Seth & Friston, 2016). This uncertainty refers to the precision with which motor and physiological states can be predicted. In this setting, negative emotions contextualise events that induce expectations of unpredictability, while positive emotions refer to events that resolve uncertainty and confer a feeling of control (Barrett & Satpute, 2013; Gu *et al.* 2013). This ties emotional states to the resolution of uncertainty and, through the biophysical encoding of precision, to neuromodulation and cortical gain control (Brown & Friston, 2012).

In summary, one can associate the valence of emotional stimuli with the precision of prior beliefs about the consequences of action. In this view, positively valenced brain states are necessarily associated with increases in the precision of predictions about the (controllable) future – or, more simply, predictable consequences of motor or autonomic behaviour. Conversely, negative emotions correspond to a loss of prior precision and a sense of helplessness and uncertainty about the consequences of action.

## What about mood?

Any hierarchical inference relies on hyperpriors. These furnish higher level predictions of the likely value of lower level parameters. From the above, one can see that important parameters are the precisions of prediction errors at high and low levels of the hierarchy (i.e. prior and sensory precision). These precisions reflect the confidence we place in our prior beliefs relative to sensory evidence. If emotional states in the brain reflect the precision of prior beliefs about the consequences of action, then distinct neuronal populations must also encode hyperpriors. In other words, short-term fluctuations in precision (i.e. emotional fluctuations) will themselves be constrained by hyperpriors encoding their long-term average (i.e. mood).

Here, we propose that mood corresponds to hyperpriors about emotional states, or confidence about the consequences of action. In other words, mood states reflect the prior expectation about precision that nuances (emotional) fluctuations in confidence or uncertainty. If emotion reflects interoceptive precision, and is biophysically encoded by neuromodulatory gain control, then this suggests that mood is neurobiologically encoded as the set-point of neuromodulator systems that determine synaptic gain control over principal cells reporting prediction errors at different levels of the interoceptive hierarchy. This set-point is the sensitivity of responses to prediction errors and has a profound and enduring effect on subsequent inference.

## When mood goes wrong

**Fig. 1.**
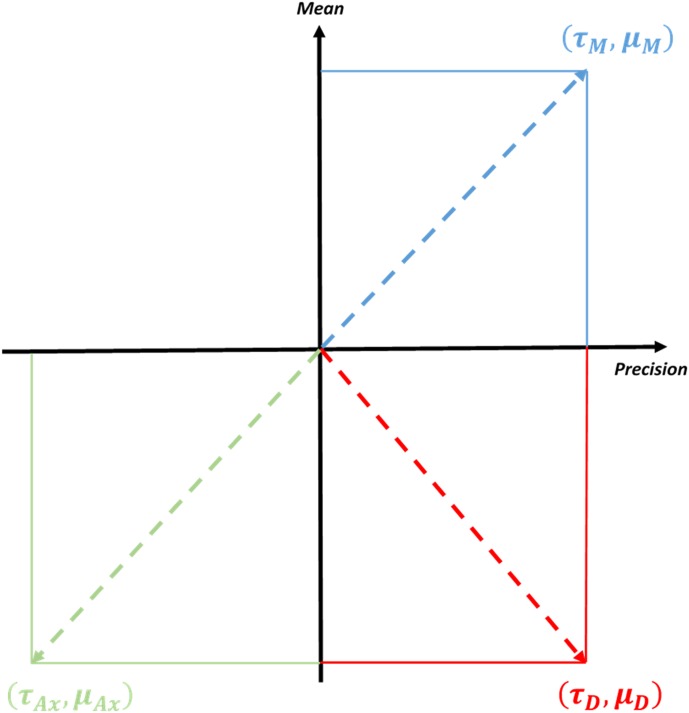
The figure shows how mood can be conceptualised according to the expected precision (*μ*) and precision of precision (*τ*) in a two-dimensional space. Here, precision *per se* corresponds to the predictability of the (prosocial, affiliative and interoceptive) world – and the two dimensions correspond to hyperpriors over precision. It is proposed that pathological changes in mood occur in the extrema of this space, as highlighted. Depression occurs when an uncertain, unpredictable outcome is predicted with high precision (red lines) resulting in a chronic, self-maintaining negative emotional state that is resistant to revision. Mania (blue lines) is characterised by an equally high precision, but with the expectation of a predictable and controllable outcome – correspondingly the environment is chronically and inappropriately labelled as such. Anxiety (green lines) is an expected unpredictability but with low precision. As such, the individual engages in behaviour designed to resolve this uncertainty but which never does. D, depression; M, Mania; Ax, anxious depression.

An interesting corollary of the above account is that mood becomes a two-dimensional construct – according to the sufficient statistics (i.e. mean or expectation and precision) of hyperpriors over interoceptive precision (Fig. 1). In this sense, we might conjecture that major depression occurs when the brain is certain that it will encounter an uncertain environment, i.e. the world is inherently volatile, capricious, unpredictable and uncontrollable. There are several concomitants of this state of affairs: if, *a priori*, prior beliefs are deemed imprecise, then the attenuation of interoceptive prediction errors will be compromised. This means that allostatic control is precluded – and is replaced by low-level homoeostatic responses (Barrett *et al.* 2016; Stephan *et al.* 2016; Peters *et al.* 2017). A corollary of this is that patients with low mood (low prior precision) should show a hypersensitivity to interoceptive cues (high sensory precision) and a failure of sensory attenuation (Badcock *et al.* 2017) – of the sort associated with stress responses (please see below).

**Fig. 2.**
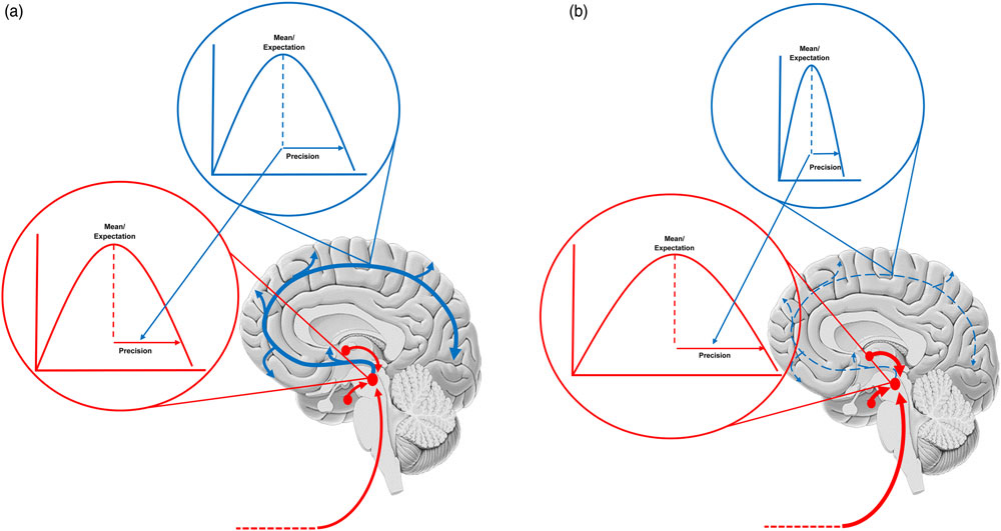
The figure shows a schematic of the neuromodulatory systems with the probability distributions the embody also displayed. Ascending projections (prediction errors) are shown in red and cortical projections (predictions of precision) are in blue. The expected precision (at different levels of the cortical hierarchy) is encoded by a tonic drive – that exerts a gain control over the red (ascending) projections. Each ascending projection conveys some newsworthy (unpredicted) information. The cortical hierarchy assembles this information (i.e. prediction errors) into an updated representation of the body and world – including its predictability. Our focus in this paper is predictions of predictability (i.e. precision) that are informed by the amplitude of prediction errors from different parts of the cortical hierarchy. (a) Shows the balance in a healthy system. Mood is liable to change with environmental fluctuations due to a precision that mediates fluctuations in synaptic gain. (b) Shows how this fails in depression. A chronically stressful environment has mandated a tonically depleted serotonin drive and the estimated precision is chronically low. This precludes precise prior beliefs (and adaptive stress reducing influences from, e.g prefrontal cortex), thereby exposing cortical updating to ascending (unattenuated) autonomic drives. It is important to note this schematic is highly simplified and that similar changes may play out in other neuromodulatory systems across other mood disorders.

Clearly, hyperpriors may be genetically encoded, although they may also change following chronic periods of intense stress (see below). This means the set-point of neuromodulator systems becomes configured to an aberrant tonic drive that is resistant to negative feedback loops that relay error messages (i.e. a loss of high-level precision that is reflected in persistently abnormal neuromodulation at the synaptic level). This may relate particularly to anhedonia (Chekroud, 2015). In this formalism, pleasure signals (bottom-up signals which increase interoceptive precision and confer a sense of control) – such as those generated by hedonic hotspots in hierarchically deep limbic circuitry (Berridge & Kringelbach) are attenuated – so feelings of pleasure are never initiated (Fig. 2). Such exquisite gain control – that normally allows for a precise repertoire of (stress reducing) behaviour – is denied to the depressed individual, who will fail to engage in (allostatic) actions that are likely to mitigate negative emotions. As precision enforces prediction-fulfilling action, maladaptive behaviours may therefore be conceptualised as an aberrant action-perception cycle that is self-reinforcing – or self-evidencing (Hohwy, 2016).

Mania may be associated with a comparable level of precision over expected emotional states but here, expectations are shifted towards positive emotions (of a secure, predictable, controllable and epistemically rich world). This means that the set-point of neuromodulator systems will be quantitatively distinct from depression; although they may become equally resistant to feedback. Accordingly, manic individuals lose the capacity to appreciate the unpredictable consequences of their actions and will engage in overconfident, high-risk behaviours (Mason *et al.* 2017). This has interesting parallels with theories of optimal control and reinforcement learning in which a link between action (e.g. pushing a button) and outcome (e.g. losing money) reinforce avoidance behaviour – a phenomenon which is absent or impaired in mania (see below) (Bach & Dolan, 2012).

The existence of mixed states can also be accounted for under this model. Note that mania and depression share an inappropriately precise prior over emotional states. As such, predictions encoded by such a precise prior – which do not lie at the extrema of mean values – could potentially manifest as mixed states, showing some manic and some depressive features. In Fig. 1 such states would lie in the intermediary region between the top and bottom right-hand corner.

Similarly, anxious depression can be described by a highly uncertain belief (hyperprior) about negative emotions (a loss of prior precision). This means the tonic drives of neuromodulator systems should be comparable to that in depression, but remain more responsive to peripheral feedback. One interesting consequence of this state, however, is a lack of action, given action is only possible when prior expectations are precise. In short, by framing mood as the hyperprior over emotional states, we can describe a wide range of abnormal mood states according to their different co-ordinates along a two-dimensional continuum.

## The existing evidence

### Neuromodulatory systems

If the account on offer is correct, we would expect an aberrant set-point for various neuromodulator systems that are specifically associated with abnormal mood states. More formally, these systems will be configured to a set-point whereby levels of stress modulators are elevated and anti-stress modulators are lowered, and receptor sensitivity is altered to ensure these levels are resistant to negative feedback from bottom-up (interoceptive) feedback from the body.

In healthy systems, mood should be affected by the valence of tightly controlled prediction errors. Recent animal work has shown that positive prediction errors (receiving more food than expected), show a strong positive correlation with dopaminergic change in the nucleus accumbens (Hart *et al.* 2014) with corresponding changes in functional brain activity in humans during a financial reward task (Rutledge *et al.* 2010). Similarly, it has been shown that signal change in the anterior insula is significantly related to the magnitude of prediction error (Bossaerts, 2010). The pharmacological manipulation of these networks was recently demonstrated where participants were given electric shocks (harms) in exchange for financial reward (gains), and offered the option of increasing the number of shocks in exchange for greater reward. It was shown that citalopram increased harm-aversion, while levodopa made individuals more likely to harm themselves than others (Crockett *et al.* 2015). This fits nicely with our notion that serotonin levels (and other neuromodulators) encode expectations about likely negative outcomes and encourage the fulfilment of these predictions through action (i.e. low levels promote behaviour with negative outcomes).

A much richer literature of neuropharmacology in mood disorders exists, and fits nicely with our theories. What is crucial is that the same systems are implicated across depression, mania and anxiety though the basal levels (expectations) and feedback sensitivity (precision) of these systems differs accordingly. Take, e.g. the hypothalamic pituitary adrenal (HPA) axis. In depression there is increased paraventricular nucleus (PVN) drive and CRH production (Raadsheer *et al.* 1994; Gao *et al.* 2013) and resistance to glucocorticoid receptor (GR) mediated negative feedback (Holsboer *et al.* 1982; Sher *et al.* 2013). We would expect that anxiety states are associated with similar basal drive but increased resistance to feedback. Indeed it has been shown that patients with post-traumatic stress disorder (PTSD) have heightened HPA axis drive originating at the amygdala (Shin *et al.* 2006) and PVN (Kasckow *et al.* 2001) and decreased inhibitory input from the hippocampus (Smith, 2005), although they show increased sensitivity to dexamethasone suppression test (Yehuda *et al.* 1993). This explains the finding of lower cortisol levels in certain testing conditions only (Meewisse *et al.* 2007). Mania is less well studied but there are reports of underactive HPA axis drive being particularly related to euphoria (Valiengo *et al.* 2012).

### The effects of environmental trauma

If the brain is an organ of inference, that attempts to reduce the surprise associated with environmental outcomes, then its synaptic activity and tonic drives should be in line with its (prior and hyperprior) expectations. Active inference attempts to construct an environment (and physiology) consistent with these expectations. However, chronic unpredictability warrants a change in reliability or precision afforded to social and physiological cues. In this regard, active inference may explain the established finding that childhood trauma poses a risk for various psychopathologies (Bernet & Stein, 1999; Heim & Nemeroff, 2001; Heim *et al.* 2008). In this context, it is important to acknowledge how different types of traumatic experiences may impact differentially on predictions in the brain. Stress of any kind induces uncertainty, though the nature of this stress may determine whether the mood state will be a depressed one or an anxious one. More chronic and less well-defined adversity – which is experienced during emotional or physical neglect – is pervasive and enduring and should result in great certainty over uncertain (interoceptive) outcomes of (affiliative or prosocial) action. As such, depression would be associated with this type of trauma. In line with this, a recent study in bipolar depression demonstrated that, despite all domains of the childhood trauma being significantly more prevalent in patients than controls, only emotional neglect predicted psychopathology (Watson *et al.* 2014). Conversely, more acute and explicit traumatic experiences would change expectations but render them highly uncertain (i.e. induce an anxiety state). This is perhaps best exemplified by the risk posed for PTSD by acute and extreme episodes of trauma (Sullivan *et al.* 2006).

One important corollary of the theory presented above is that risk of mood disorders is intimately tied to emotional trauma only. This accords with the findings from recent studies (Watson *et al.* 2014) though not others. Instead, it is proposed that (barring brain damage) physically traumatic events facilitate the onset of mood disorders through the subjective feelings that contextualise them. One other interesting consequence of the active inference formulation is that, as mood refers to hyperpriors over interoceptive states, it is non-specific to different types of environmental fluctuations; i.e. their particular content. This means predictable environmental outcomes elsewhere can act as a buffer against the detrimental effects of trauma (Southwick & Charney, 2012). This has clear and important ramifications for preventive strategies – and may also explain why traumatic experiences are not invariably associated with the onset of psychopathology despite biologic effects (Carpenter *et al.* 2007).

### The emergence of epigenetics

If environmental factors can induce conformational changes in the set-point of neurobiological systems to precipitate onset of mood disorders, then some explanatory mechanism is required. Epigenetics has emerged as a major field of enquiry in recent years, specifically in relation to methylation of the NR3C1 gene, which encodes GR (Nantharat *et al.* 2015; Smart *et al.* 2015; Palma-Gudiel *et al.* 2016). Crucially, GRs are found in the hippocampus and amygdala (Morimoto *et al.* 1996); regions that send descending predictions to the paraventricular nucleus (Herman *et al.* 2002). A loss of sensitivity to circulating cortisol levels in the amygdala and hippocampus may be the neurophysiological correlates of aberrant hyperpriors that set the neuromodulatory tone for amplification and attenuation of the PVN. Although speculative, this provides a potential framework that can be modelled in terms of active inference and is entirely consistent with theories based upon allostasis (McEwen, 2000; Radley *et al.* 2011; Braithwaite *et al.* 2015).

Our ideas also explain why epigenetic variability tends to occur at critical periods of development (Heim & Binder, 2012). In this regard it is important to remember that error signals can only be attenuated in states of high prior precision (confidence in the consequences of behaviour). Crucially, this certainty can only be inherited from a stable environment experienced over time, and so epigenetic alteration in neuromodulatory systems is more likely to occur when systems experience new or unpredictable environments and thus expect a higher degree of uncertainty. The most obvious time when this would occur is the immediate neonatal and infancy period when biological systems have almost no prior experience. The set-point of these systems, at this time, encodes an imprecise (i.e. flat or uninformative) prior that is waiting to be informed through experience. This conforms to an elegant study by Weaver *et al.* (2004) who showed a rapid increase in DNA methylation in rodents the day after birth. However, they also showed that methylation rates rapidly declined in rodents who were maternally groomed and nurtured while rates remained elevated in neglected pups (Weaver *et al.* 2004). This implicates a role of maternal care in establishing an appropriate physiological set-point and ensuring it is precise enough to resist some form of future stress challenge. It also shows that adversity experienced in *early* life is critically important in establishing vulnerability towards onset of mood disorders.

### Psychological theories

Psychological theories of mood are important and remain a challenge for most biological accounts. Perhaps the best model to date is *learned helplessness* (Seligman, 1975). We have characterised depression by inappropriately high precision in the negative consequences of action for the individual's internal states. Accordingly this results in failure by the individual to engage with potentially positive outcomes of action. However, as discussed above, high precision also facilitates allostasis in order to preserve current states and so not only would depression result in failure to attend to positive stimuli but it would result in active inference to preserve the depressed state resulting in the behaviour that we associate with learned helplessness.

A more recent and promising psychological model of depression stems from the demonstration of attentional bias in patients towards negative facial expressions (Duque & Vázquez, 2015). Harmer *et al.* have thus proposed that a negative emotional bias is a core feature of depression and is the psychological target of antidepressant medication (Harmer *et al.* 2009; Harmer & Cowen, 2013) and correspondingly that modulating attentional bias can provide therapeutic benefit (Browning *et al.* 2012). Active inference requires precise coding so interoceptive information that is inconsistent with the current state can be attenuated, as such, in depression, we would expect sensory attenuation away from positive stimuli and greater attention towards negative stimuli – in line with psychological expectations that are biologically encoded.

Much experimental work has also shown an increased response to rewarding stimuli in bipolar disorder, which can be accounted for by our theory in which manic patients expect a pleasurable outcome (i.e. a reward) from their actions – even if this is highly unlikely. This has been demonstrated using self-report measures of behavioural drive (Van der Gucht *et al.* 2009) and response time analyses to financial reward cues (Singh *et al.* 2013). Accordingly patients also show a failure to learn from punishing cues (Mueller *et al.* 2010). Conversely, depressed patients show a reward hyposensitivity in line with overly precise prior prediction of a negative outcome and subsequent aversion to positive bottom-up (interoceptive) signals (Eshel & Roiser, 2010).

## Future work

If the functional anatomy of mood involves alterations in tonic neuromodulatory drives, then it will affect communication between higher and lower levels of the extended interoceptive system. As such, any functional brain investigations in mood disorders must be capable of quantifying effective connectivity between relevant networks and evaluating how this connectivity is modulated by external factors. Dynamic causal modelling is well placed in this regard (Friston *et al.* 2003) and could prove a fruitful tool for further investigation – specifically in examining the task-dependent coupling between hierarchical levels of neuromodulator control (Schlosser *et al.* 2008; Lu *et al.* 2012; Radaelli *et al.* 2015; Vai *et al.* 2015; Vai *et al.* 2016).

Given the discussions above, we would expect not only differences in effective connectivity along neuromodulatory axes, but also differences in the way this connectivity is modulated according to external stimuli. Very few studies have been conducted along these lines but it is interesting to note a handful that have. Sladky *et al.* showed that patients with social anxiety disorder demonstrated increased activation in orbitofrontal cortex and amygdala when viewing emotional faces (Sladky *et al.* 2012), but furthermore, in controls this network shows top-down modulation – which is reversed in patients – orbitofrontal cortex drives greater activation in amygdala (Sladky *et al.* 2015). This fits nicely with our theories; where pathological anxiety is associated with an expectation of emotional negative environment (increased tonic activity in the limbic–prefrontal network) but also with a complete loss of precision and failure to dampen ascending information (loss of top-down inhibitory control). Prefrontal–amygdala connectivity in depressed patients has also been investigated in two studies, showing reduced top-down dorsolateral prefrontal modulation of amygdala response to negative images in bipolar depression (Radaelli *et al.* 2015) with a converse pattern of increased orbitomedial–amygdala connectivity when viewing positive faces (de Almeida *et al.* 2009). This is very much in line with our arguments presented above, but the imperative remains for further studies that can fully quantify neurobiologically coded expectations and precisions across different axes.

The perspective afforded by mood as a hyperprior suggests a separation of timescales in terms of responding to prediction errors. An adaptive response to a volatile environment in which the amplitude of prediction errors is, itself, on average high would suggest a mood-lowering reduction in the estimated precision of prediction errors. This is something that could, in principle, be tested experimentally using a mood induction paradigm predicated on experimentally induced prediction errors. One interesting way of achieving this may be measuring gaze duration in mood congruent and incongruent ambiguity resolving contexts, during word reading tasks.

Our theoretical account of mood may also be useful in informing molecular studies by hypothesising how alterations in interoceptive computation might play out biologically, and how this can be manipulated therapeutically. Take the example of depression. As discussed this state corresponds to the very precise expectation of a negatively valenced environment, which allows resistance to contradictory (positive) information and ensures behaviour in line with the depressed state. We are therefore presented with two possible avenues for treatment:

(1) Alter the expectation of an uncertain and uncontrollable body (or world)
(2) Alter the body (or world) to enable a revision of expected uncertainty.

These broadly concord with current treatment approaches of which the first the most widely used via pharmacological manipulations of neuromodulation. If depression – and other mood disorders – are the result of a computational pathology then no single neuromodulator system will be implicated in every patient; possibly reflecting the vast number of treatment-resistant patients and the failure to find a consistent biomarker. Higher level processing, as considered here, is the result of an array of ascending inputs and so the same computational pathology may be manifest by any pathophysiology that involves neuromodulatory systems. This speaks to figuring out ways to tailor pharmacological interventions to better match the needs of individual patients. One recent and novel study has employed machine-learning techniques to predict treatment outcome in clinical trials (Chekroud, 2015) and represents a potentially important approach; however, it may be possible to go one step further and replace the search for a biomarker with a computational signature (Nitsche *et al.* 2010; Huys *et al.* 2011; Barch *et al.* 2012; Montague *et al.* 2012; Wang & Krystal, 2014).

## Conclusions

Much evidence over recent decades has converged on the idea that the brain is in the game of predicting its sensorium and working to minimise the difference between these predictions and actual sensory input. Further evidence suggests emotional states reflect the precision associated with neurobiological predictions over interoceptive states. In this paper, we have extended this formalism to a further level of the hierarchy and suggested mood acts as a hyperprior over emotional states. This notion has gained traction as an explanation for autism and schizophrenia in the exteroceptive (perceptual) domain (Lawson *et al.* 2014; Corlett, 2017; Krystal *et al.* 2017). We have explored the evidence for this theory and suggested how it might inform further research.
